# PDCD10 promotes proliferation, migration, and invasion of osteosarcoma by inhibiting apoptosis and activating EMT pathway

**DOI:** 10.1002/cam4.5025

**Published:** 2022-07-18

**Authors:** Ke Xu, Wenchao Fei, Ziqi Huo, Shuoer Wang, Yinghua Li, Gong Yang, Yang Hong

**Affiliations:** ^1^ Department of Orthopedics, The Fifth People's Hospital of Shanghai Fudan University Shanghai China; ^2^ Shanghai Clinical Research Center for Aging and Medicine Shanghai China; ^3^ Center of Community‐Based Health Research Fudan University Shanghai China; ^4^ Department of Musculoskeletal Surgery Fudan University Shanghai Cancer Center Shanghai China; ^5^ Department of Nuclear Medicine Fudan University Shanghai Cancer Center Shanghai China; ^6^ Department of Oncology, Shanghai Medical College Fudan University Shanghai China; ^7^ Central Laboratory, The Fifth People's Hospital of Shanghai Fudan University Shanghai China; ^8^ Cancer Institute Fudan University Shanghai Cancer Center Shanghai China

**Keywords:** apoptosis, EMT, osteosarcoma, PDCD10, therapy

## Abstract

**Background:**

Osteosarcoma, a common primary malignant tumor, occurs in children and adolescents with a poor prognosis. The current treatment methods are various, while the five‐year survival rate of patients has not been significantly improved. As a member of the programmed death factor (PDCD) family, programmed death factor 10 (PDCD10) plays a role in regulating cell apoptosis. Several studies of PDCD10 in CCM and cancers have been reported before. However, there are no relevant research reports on the effects of PDCD10 on osteosarcoma.

**Methods:**

We used bioinformatics analysis, IHC, and clinical data to confirm the expression of PDCD10 and its correlation with prognosis in osteosarcoma. Then, we used shRNAs and cDNA to knock down or overexpress PDCD10 in U2OS and MG63 cell lines. A series of function assays such as CCK8, Wound healing test, Plate cloning formation assay, and Transwell were done to confirm how PDCD10 affects osteosarcoma. Animal assays were done to confirm the conclusions in cell lines. At last, WB was used to measure the protein expression levels of apoptosis and the EMT pathway.

**Results:**

PDCD10 was highly expressed in patients with osteosarcoma and correlated with prognosis; PDCD10 knockdown inhibited osteosarcoma growth, proliferation, migration, and invasion; PDCD10 overexpression promoted osteosarcoma growth, proliferation, migration, and invasion. In vivo experiments confirmed the conclusions in cell lines; PDCD10 inhibited apoptosis and activated the EMT pathway.

**Conclusions:**

In this study, it was found that PDCD10 was highly expressed in patients with osteosarcoma, and it was closely related to patient prognosis. PDCD10 inhibited tumor cell apoptosis and promoted tumor progression by activating the EMT pathway. These findings may provide a potential target for gene therapy of osteosarcoma.

## Graphical abstract

In this study, it was found that PDCD10 was highly expressed in patients with osteosarcoma, and it was closely related to patient prognosis. PDCD10 inhibited tumor cell apoptosis and promoted tumor progression through activating EMT pathway. These findings may provide a potential target for gene therapy of osteosarcoma.

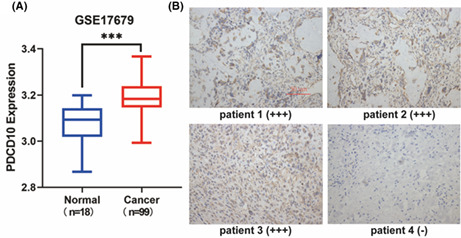

## 1 INTRODUCTION

Osteosarcoma is a malignant tumor seriously endangering adolescents^1^ due to the extremely poor prognosis and high invasive and metastatic properties.^2^ Meanwhile, apart from leukemia and lymphoma, osteosarcoma is the most common malignant tumor in adolescents.^3^ At present, clinical treatment mainly includes surgery,^4^ radiation,^5^ neoadjuvant chemoradiotherapy, drug therapy,^6^ and other methods.^7^ The comprehensive use of existing treatment techniques can improve the five‐year survival rate of patients, but some patients still suffer from metastasis and recurrence.^8^ The poor prognosis drives us to explore more advanced treatment methods. For example, anti‐angiogenesis drugs,^6^ viral vector gene therapy, and immunotherapy have been initially applied. It is crucial to determine the molecular mechanisms related to the growth and metastasis of osteosarcoma, which could find new treatments for osteosarcoma.

Programmed cell death (PDCD) is a class of proteins that are associated with tumor development and are highly conserved in evolution.^9^ The PDCD family consists of multiple members, and it is widely distributed in various human tissues and cells, with the main function of regulating apoptosis. Programmed cell death 10 (PDCD10) was found in human TF‐1 cells and was originally called TF‐1 cell apoptosis‐related gene 15 (TFAR15) is an important factor regulating cell apoptosis.^10^ It was also called CCM3.^11^ In addition, PDCD10 can not only inhibit tumor cell proliferation but also prevent cell apoptosis under different conditions.^12^, ^13^ At present, there have been studies in CCM,^14^, ^15^ ovarian cancer,^16^ breast cancer,^17^ prostate cancer,^18^ drug resistance,^19^ and brain tumors^20^ about PDCD10. However, there are no relevant research reports on the effects of PDCD10 on osteosarcoma.

Epithelial‐mesenchymal transition (EMT) promotes a biological process by which epithelial cells are transformed into cells with a mesenchymal phenotype through a specific program.^21^ Therefore, EMT plays an important role in the occurrence and development of many tumors, with the main features of reducing the expression of cell adhesion molecules (such as E‐cadherin) and transforming the cytokeratin into a vimentin‐based cytoskeleton.^22^ EMT is an important biological process in which malignant tumor cells from epithelial cells acquire the ability to migrate and invasion.^23^ Therapeutic methods targeting the key molecules of EMT are important methods for the treatment of tumors. Apoptosis refers to a basic biological phenomenon of cells, ad it is strictly controlled by many genes such as the Bcl‐2 family, caspase family, oncogenes such as C‐myc, tumor suppressor gene P53, and so on.^24^ Under the action of tumors, immune system diseases, and drugs, the normal apoptosis program of cells can be affected.

In this study, it was proved that PDCD10 can inhibit cell apoptosis and activate the EMT pathway to promote the progress of osteosarcoma, which will provide new targets for future therapy.

## 2 MATERIALS AND METHODS

### 2.1 Microarray data

The GSE17679 was downloaded from the GEO database (http://www.ncbi.nih.gov/geo). The extracted data were normalized by log2 transformation in R software (version 3.4.1). Probes were converted to gene symbols according to the annotation information of the normalized data in the platform. Probes matching multiple genes were removed from these datasets. The average expression value of the gene measured by multiple probes was calculated as the final expression value. The result of the data preprocessing was assessed by a boxplot. The PCA plot was drawn to illustrate the samples before and after the batch effect.

### 2.2 Patient recruitment

In this study, we randomly selected 38 patients with osteosarcoma who were admitted to the hospital from 2015 to 2020. Among these patients, 17 males and 21 females, aging from 17 to 64, received surgical operation in the hospital, and the postoperative pathological results confirmed the diagnosis of osteosarcoma. The tumor sites mainly included tibia, femur, and vertebrae. Conventional chemotherapy was performed after the operation. We followed up for 5 years by outpatient and telephone calls to track the patient's treatment efficacy and 5‐year survival rate. All patients were fully informed and signed informed consent.

### 2.3 Immunohistochemistry(IHC)

Briefly, 5 μm sized paraffin‐embedded tissue sections were de‐paraffinized with xylene and endogenous peroxidase activity was quenched with 3% H2O2 in methanol for 30 minutes. Tissue sections were dehydrated through graded alcohols and subjected to antigen retrieval using 10 mM sodium citrate. Sections were washed with TBST and then blocked with 5% BSA for 1 h. Slides were incubated with the primary anti‐PDCD10 antibody (A17094; ABclonal; 1:100) diluted with TBST. Slides were then washed for 5 minutes 3 times in TBST and incubated for 1 h with anti‐rabbit secondary antibody (Cell Signaling Technology; 1:2000) diluted with TBST. Then, slides were incubated with DAB (Sigma) and immediately washed under tap water after color development. Slides were then counter‐stained with hematoxylin. Slides were mounted with DPX and were then observed under the microscope (Leica).

### 2.4 Cell culture and transfection

Two cell lines, namely U2OS and MG63, were selected and cultured in DMEM containing 10% FBS and 1% PS in a 37°C incubator with 5% carbon dioxide. Stable transfection cell lines were constructed and infected with lentivirus and screened with puromycin/hygromycin.

### 2.5 Reagents and antibodies

Dulbecco's Modified Eagle Medium (DMEM) and Fetal bovine serum (FBS) were purchased from Gibco (11,965,092; 26,140,079). The primary antibodies used were anti‐PDCD10 (A17094; ABclonal; 1:500), −caspase3 (A0214; ABclonal; 1:500), −capase9 (A2636; ABclonal; 1:500), ‐BCL‐2 (ab32124; abcam; 1:1000), ‐BAX (ab32503; abcam; 1:1000), ‐FLAG (TAX159Ge22; Cloud‐Clone Corp; 1:1000), −β‐actin (ab8226; abcam; 1:1000), and ‐GAPDH (G9295; Sigma; 1:1000). The secondary antibodies were goat anti‐rabbit and ‐mouse antibodies (7074, 4410; Cell Signaling Technology; 1:2000, 1:2000).

### 2.6 Plasmid construction

The main plasmids, including psPAX2, pmD2.G, pLKO.1‐puro, pLKO.1‐hygro were used for the lentivirus system. Three PDCD10 shRNAs (Sh1: 5′‐GCTGATGATGTAGAA GAGTAT‐3′, Sh2: 5′‐CCAGATGAGATCAATGACAGA‐3′, Sh3: 5′‐CGTAAGTGCCAACCGACTAAT‐3′) targeting human PDCD10 mRNA were inserted into pLKO.1‐puro. The full‐length DNA coding sequence of PDCD10 was inserted into pLKO.1‐hygro.

### 2.7 Quantitative real‐time polymerase chain reaction (qRT‐PCR)

According to the operation procedure, TRIZOL was used to collect RNA, and a reverse transcription kit was used to obtain cDNA, and then primers and reaction buffer were added forward and reversed to start the reaction. At last, the data were obtained for analysis after the reaction was completed in the machine.

### 2.8 Western blot (WB)

Cells were lysed with RIPA solution containing 1% PMSF, the protein was measured with BCA reagent, and protein samples were prepared with 5X loading. After SDS gel electrophoresis, the protein was transferred to the PVDF membrane. PVDF membrane was blocked with 5% skim milk at room temperature for 1–2 h and then incubated with the primary antibody for 14 h at 4°C. After washing the membrane 3 times, it was incubated with the secondary antibody at room temperature for 1–2 h. After washing the membrane 3 times, the protein expression levels were detected by using the enhanced chemiluminescence reagents.

### 2.9 Wound healing test

U2OS and MG63 cells were digested and seeded into six‐well plates, and each cell was replicated in triplicate. After the cells were full, a 1 ml pipette tip was used to cut in the middle, and the cells were washed with 1X PBS. Then DMEM (without FBS) was added, and pictures were taken under a microscope at 0 h and 24 h.

### 2.10 CCK‐8

The CCK‐8 kit was bought from 7sea biotech and applied to evaluate the effects of PDCD10 on cell proliferation of U2OS and MG63 cells by the absorbance respectively. U2OS and MG63 cells were digested and seeded at 5000 cells per well respectively into 96‐well plates with five replicates. After culturing for 0, 24, 48, and 72 h, 10 μl CCK‐8 reagent was mixed with 100 μl fresh medium and added to each well. Then the plates were incubated for 2 h at 37°C to detect the absorbance of 450 nm. At last, the data were obtained for analysis.

### 2.11 Plate cloning formation assay

U2OS and MG63 cells were digested and seeded into six‐well plates with 500 cells per well, and each cell was replicated in triplicate. After 10 days, they were fixed in 4% paraformaldehyde, stained with crystal violet, washed with 1X PBS, and photographed as last.

### 2.12 Transwell

Transwell 24‐well plates were purchased from Corning. To perform the transwell assay, we firstly used the Matrigel to coat the upper chamber of the well. Cells were digested and counted. A total of 600 μl DMEM containing 10% FBS was added to the lower chamber, 150 μl of serum‐free DMEM was added to the upper chamber, and 50,000 cells were added to the upper chamber in each well. Transwell 24‐well plates were incubated in a 37°C cell incubator, and cells were fixed in 4% paraformaldehyde after 24 h, stained with crystal violet, washed with 1X PBS, and pictures were taken under a microscope at last.

### 2.13 Animal assay

4‐week‐old Balb/c‐nu mice were used, each mouse was injected with 5 million cells, suspended in 1X PBS subcutaneously in the left armpit, and sacrificed after 4 weeks by spinal dislocation and photographed. Finally, tumors were taken to measure tumor size and weight.

### 2.14 Data analysis

All experimental data were analyzed by GraphPad Prism 9.0, and pictures were processed and integrated by Photoshop (PS) and Adobe Illustrator (AI). All data were expressed as the mean ± SD. T test and one‐way/two‐way analysis of variance (ANOVA) were used for statistical differences. *p* < 0.05 was considered to be statistically significant.

### 2.15 Ethical statement

The approval of this study was authorized by the Fifth People's Hospital of Shanghai, Fudan University, China. Animals were sacrificed according to specified standards. Clinical samples were used with the permission of the patients.

## 3 RESULTS

### 3.1 High expression of PDCD10 in osteosarcoma

**FIGURE 1:**
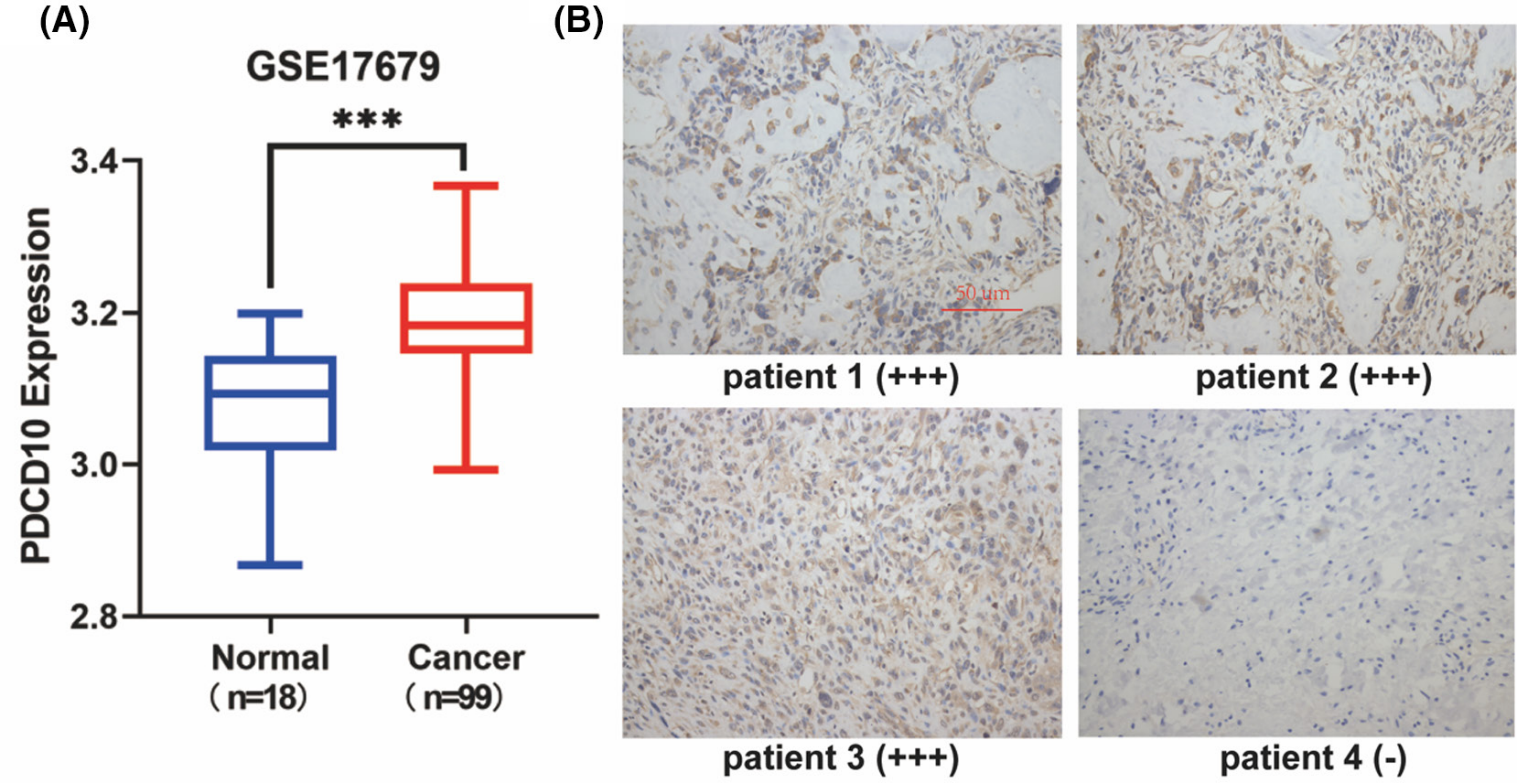
PDCD10 was highly expressed in patients with osteosarcoma. (A) Expression of PDCD10 in normal and cancer, data from GSE17679 database. Normal (*n* = 16), Cancer (*n* = 99). *p < 0.05, **p < 0.01, ***p < 0.001. (B) Patients1‐3 were immunohistochemically positive, and patient4 was immunohistochemically negative. Scale bar: 50 μm.

To explore the potential role PDCD10 played in osteosarcoma, the GSE17679 database, including normal (*n* = 18), and cancer (*n* = 99), was analyzed, and the significant expression of PDCD10 in osteosarcoma was found (Figure 1A). Even if there were reports in other tumors before,^16^, ^17^, ^18^ it was still unknown to osteosarcoma. We wondered if PDCD10 could influence the progress of the osteosarcoma.

### 3.2 The overexpression of PDCD10 in osteosarcoma and the association between osteosarcoma and clinical prognosis

Totally, 38 patients with osteosarcoma who underwent the operation admitted to our hospital from 2015 to 2020 were included, with 17 males and 21 females. As shown in Figure 1B, patients 1–3 were immunohistochemically positive and patient 4 was immunohistochemically negative. As a result, immunohistochemistry (IHC) staining displayed that 86.84% of patients were positive for PDCD10, and 33.33% of those passed away in 5 years. It was supposed that PDCD10 can deteriorate osteosarcoma.

### 3.3 The establishment of stable PDCD10 knockdown cell lines

**FIGURE 2:**
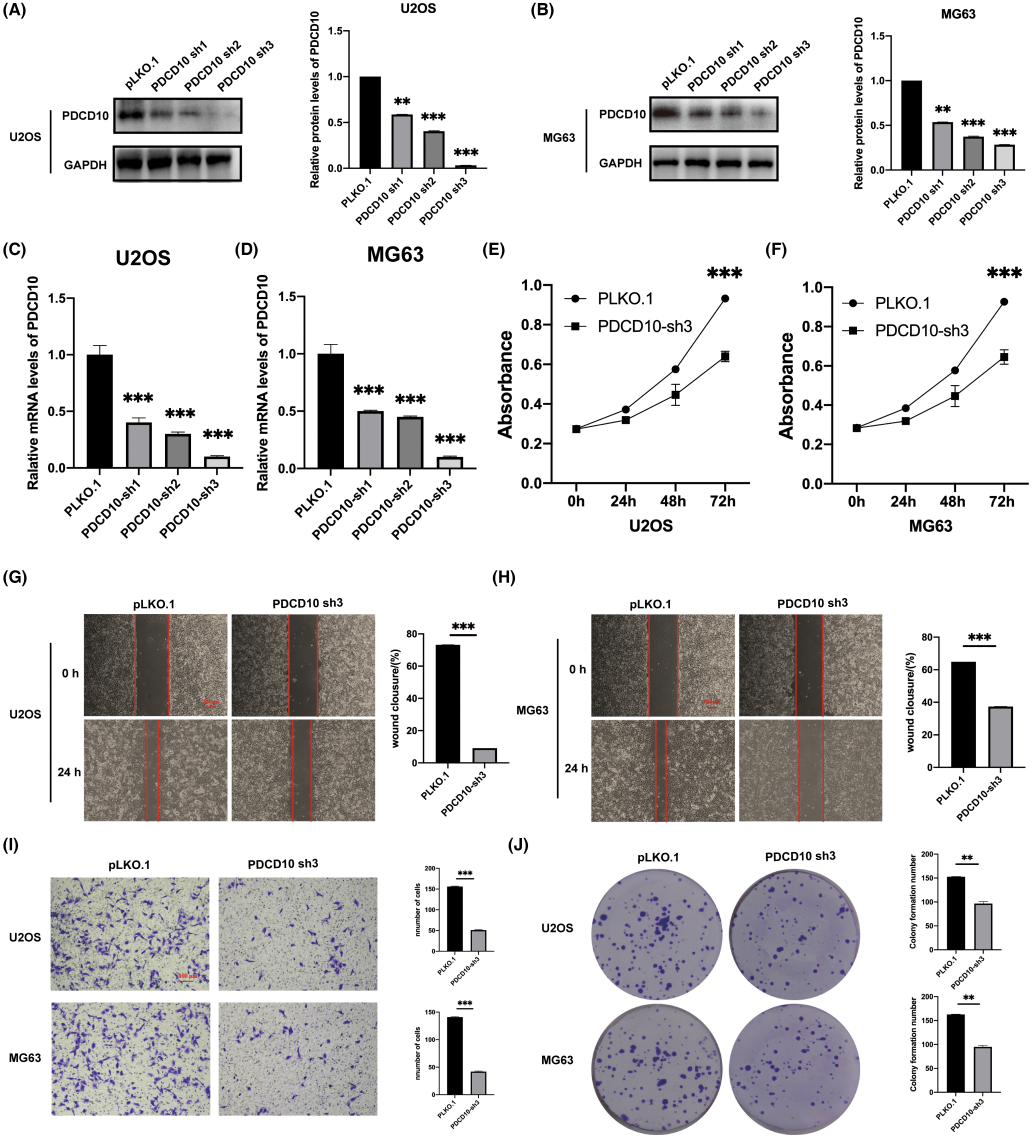
PDCD10 knockdown inhibited osteosarcoma growth, proliferation, migration, and invasion. (A, B) PDCD10 knockdown by three shRNAs in U2OS and MG63 cell lines, protein expression detected by western blot. (C, D) The mRNA levels of PDCD10 in U2OS and MG63 cell lines were detected by qRT‐PCR. (E, F) PDCD10 knockdown inhibited cell growth in U2OS and MG63 cell lines. (G, H) PDCD10 knockdown inhibited cell migration in U2OS and MG63 cell lines. Scale bar:200 μm. (I) PDCD10 knockdown inhibited cell invasion in U2OS and MG63 cell lines. Scale bar:100 μm. (J) PDCD10 knockdown inhibited cells proliferation in U2OS and MG63 cell lines. Values are expressed as means±standard deviations. *p < 0.05, **p < 0.01, ***p < 0.001.

To explore the influence of PDCD10 knockdown on osteosarcoma, U20S and MG63 cell lines were used to establish stable PDCD10 knockdown cell lines via lentiviral infection. Three shRNAs targeting PDCD10 were used. In Figure 2A,B, from the protein expression results of PDCD10 by WB and the mRNA expression results of PDCD10 by qRT‐PCR(Figure 2C,D), low expression of PDCD10 was obviously observed in both levels of protein and mRNA compared with pLKO.1 (control group).

### 3.4 In vitro experiments to testify the suppression of functions like proliferation, migration, and invasion to osteosarcoma with PDCD10 knockdown

To clarify the effects of PDCD10 on osteosarcoma, a series of experiments on PDCD10 sh3 were carried, which was the best PDCD10 knockdown cell line. As shown in Figure 2E,F,G,H,J, the growth of PDCD10 knockdown cells was slowly verified by CCK8, wound healing assay showed that PDCD10 knockdown inhibited cell migration, and plate cloning assay showed that PDCD10 knockdown inhibited colony formation. In addition, as shown in Figure 2I, the ability of invasion decreased after PDCD10 knockdown.

### 3.5 The establishment of PDCD10 overexpression cell lines

**FIGURE 3:**
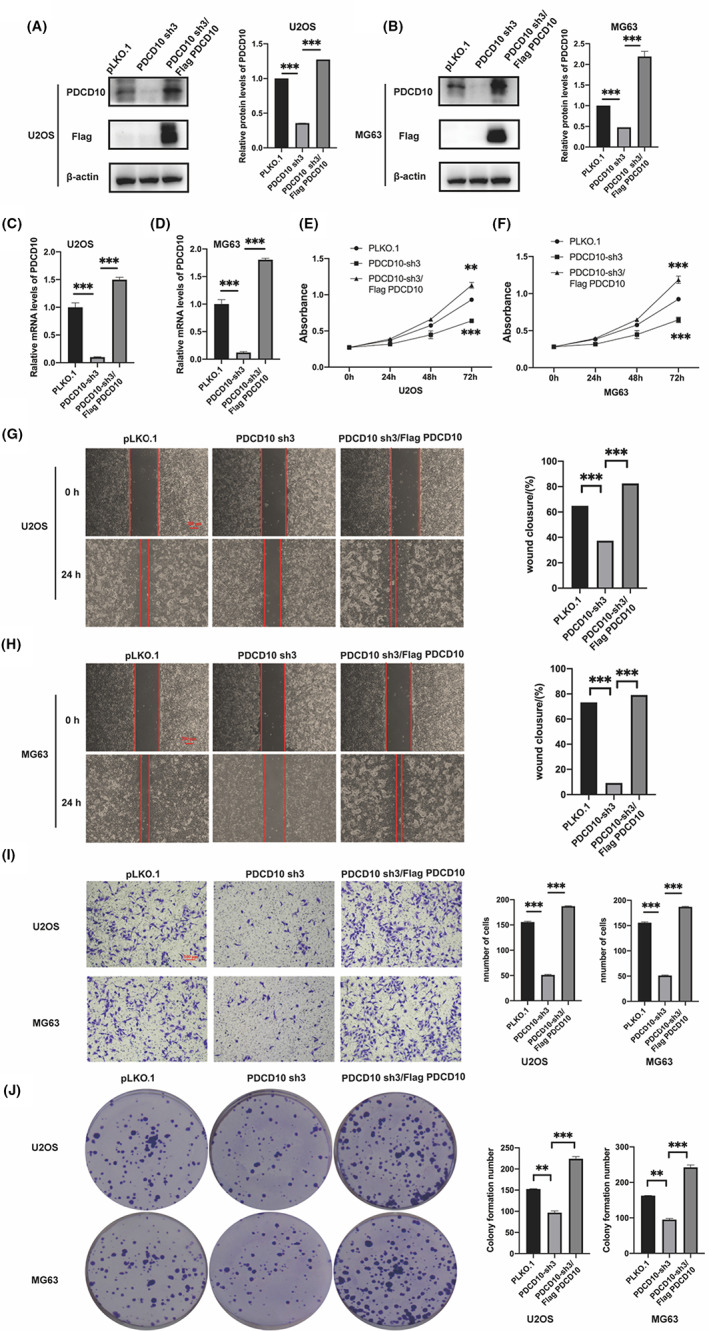
PDCD10 overexpression promoted osteosarcoma growth, proliferation, migration, and invasion. (A, B) PDCD10 overexpression by cDNA in U2OS and MG63 cell lines, protein expression detected by western blot. (C, D) The mRNA levels of PDCD10 in U2OS and MG63 cell lines were detected by qRT‐PCR. (E, F) PDCD10 overexpression promoted cell growth in U2OS and MG63 cell lines. (G, H) PDCD10 overexpression promoted cell migration in U2OS and MG63 cell lines. Scale bar:200 μm. (I) PDCD10 overexpression promoted cell invasion in U2OS and MG63 cell lines. Scale bar:100 μm. (J) PDCD10 overexpression promoted cell proliferation in U2OS and MG63 cell lines. Values are expressed as means±standard deviations. *p < 0.05, **p < 0.01, ***p < 0.001.

To thoroughly testify the influences of PDCD10 on osteosarcoma, PDCD10 knockdown U2OS and MG63 cell lines were used to establish stable PDCD10 sh3/Flag‐PDCD10 cell lines via lentiviral infection. PDCD10 cDNA containing Flag label was used. According to Figure 3A,B,C,D, the expression of both levels of protein and mRNA in PDCD10 overexpressed cells was significantly higher than that in the group PDCD10 sh3 (control group) via WB and qRT‐PCR.

### 3.6 In vitro experiments to testify the promotion of functions like proliferation, migration, and invasion of osteosarcoma with PDCD10 overexpression

In this part, pLKO.1, PDCD10 sh3 and PDCD10 sh3/Flag PDCD10 cell lines were used to prove the effects of PDCD10 repeatedly on osteosarcoma. The accelerated proliferation, migration of PDCD10 overexpressed cells were presented in CCK‐8, plate cloning assays, and wound healing, as shown in Figure 3E,F,G,H,J. Moreover, from Figure 3I, the ability of invasion had increased with the overexpression of PDCD10.

(*Notice:In order to ensure the accuracy of the experiments, in vitro experiments were performed at the same time after the cell lines were all constructed. In Figure 3, group pLKO.1 and group PDCD10 sh3 were set as control groups to compare with group PDCD10 sh3/Flag PDCD10. In order to ensure the consistency of the figures, they were as same as those in Figure 2.)

### 3.7 In vivo experiments to confirm the conclusions above

**FIGURE 4:**
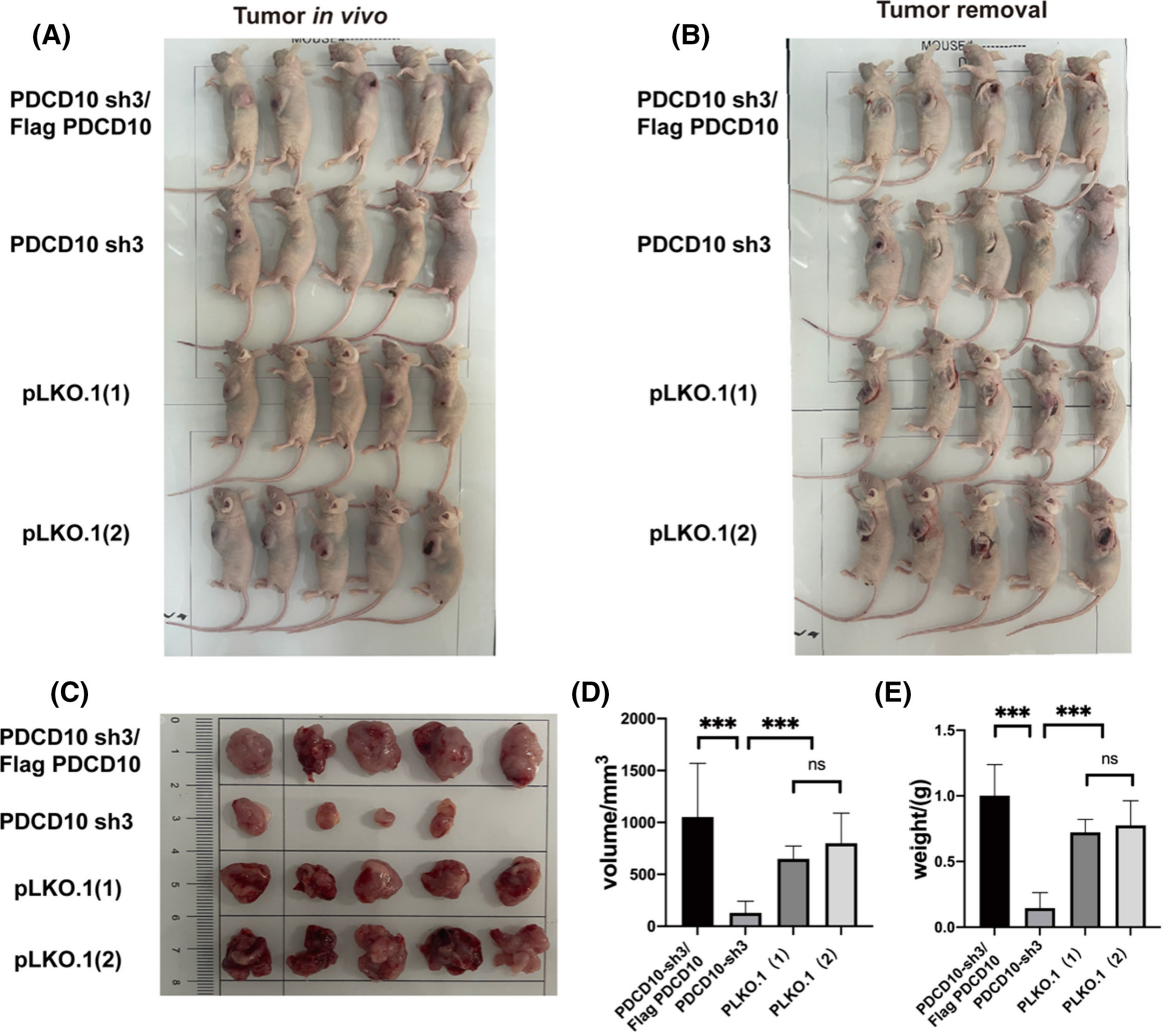
In vivo experiments to confirm the conclusions in cell lines. (A) Four groups Balb/c‐nu mice (male), tumor in vivo. (B) Four groups Balb/c‐nu mice (male), tumor removal. (C) Four groups Balb/c‐nu mice (male), tumor. (D) Tumor overexpressing PDCD10 significantly increased in size. (E) Tumor overexpressing PDCD10 significantly increased in weight. Values are expressed as means±standard deviations. *n* = 5, *p < 0.05, **p < 0.01, ***p < 0.001.

We injected subcutaneously 200 μl PBS with 5 * 10^6^ cells into the left armpit of Balb/c‐nu mice (4 week, male). Pre‐experiments showed that the U20S cell lines had low efficiency of tumorigenesis, while the MG63 was the opposite. As a result, we established the MG63‐bearing mice. As shown in Figure 4, there were no differences between the two groups of pLKO.1 (1) and pLKO.1 (2), and the error caused by the operation was excluded. The sizes and weights of the tumors were significantly reduced in the PDCD10 sh3 group compared with the pLKO.1 (1) and pLKO.1 (2) groups, while increased in group PDCD10 sh3/Flag PDCD10 compared with the group PDCD10 sh3.

### 3.8 Apoptosis inhibition and activation of the EMT pathway caused by the overexpression of PDCD10

**FIGURE 5:**
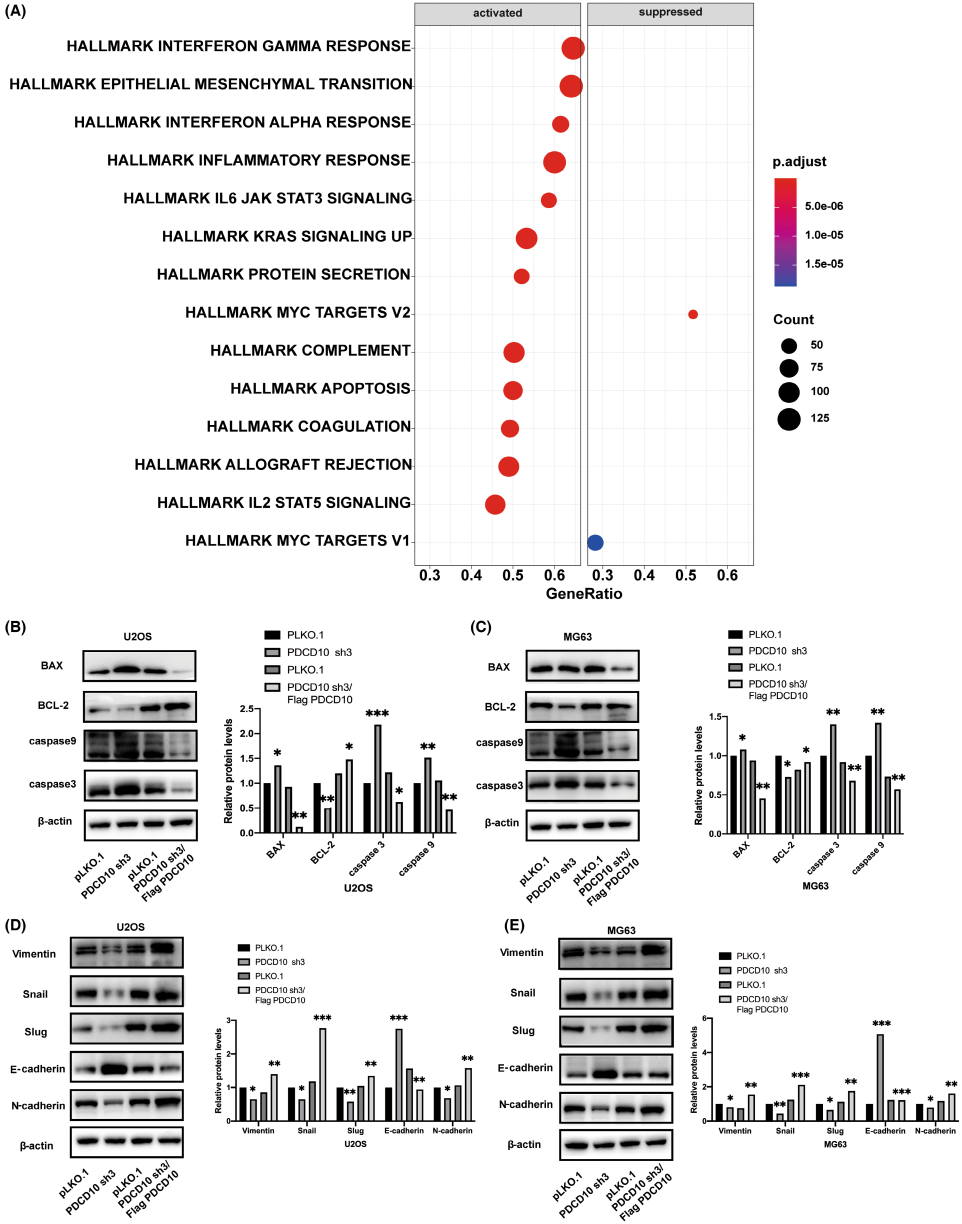
Apoptosis inhibition and activation of the EMT pathway. (A) Potential signaling pathways predicted by bioinformatics analysis, data from GSE17679 database. (B, C) PDCD10 overexpression could inhibit cell apoptosis and PDCD10 knockdown could promote cell apoptosis. (D, E) PDCD10 overexpression could activate the EMT pathway and PDCD10 knockdown could silence the EMT pathway. Values are expressed as means±standard deviations. *p < 0.05, **p < 0.01, ***p < 0.001.

In order to explore how PDCD10 affects the proliferation and invasion of osteosarcoma, we analyzed relevant pathways by analyzing the GSE17679 database (Figure 5A). The WB results indicated that the overexpression of PDCD10 could inhibit apoptosis (Figure 5B,C) and promote the proliferation, migration, and invasion of osteosarcoma by activating the EMT pathway (Figure 5D,E). PDCD10 knockdown was the opposite.

## 4 DISCUSSION

At present, more researches are conducted on tumor. Osteosarcoma is a malignant tumor^25^ that seriously endangers adolescents, with a high degree of malignancy, strong invasiveness and metastasis, and a high mortality rate within 5 years. The diagnostic methods of clinical features mainly include clinical symptoms, signs, X‐ray, CT, MRI, PET‐CT,^26^ and pathological biopsy.^27^ Its typical symptoms are pain and swelling at the tumor site that is caused by tumor tissue infiltration and osteolysis.^28^, ^29^ Currently, there are various treatments for osteosarcoma such as local tumor resection, drug therapy, chemotherapy, radiochemotherapy, neoadjuvant radiochemotherapy, immunotherapy,^30^ and gene therapy.^7^, ^31^, ^32^ In addition to some of the above‐mentioned treatment methods, nanomaterials have also been gradually used in the diagnosis and treatment of osteosarcoma, which displayed their unique advantages.^33^ Although there are many available treatments, the results are far from satisfactory and more effective methods need to be constantly found. Many signaling pathways play an important role in the occurrence and development of tumors including NF‐kB,^34^ PI3K/AKT/mTOR,^35^ MAPK,^36^ JAK–STAT,^37^ TGF‐β,^38^ Notch,^39^ and Hippo.^40^ Among them, EMT is particularly important for tumor metastasis and migration because it can reduce the expression of cell adhesion molecules, convert the keratin cytoskeleton into vimentin, and enable cells to acquire some of the characteristics of mesenchymal stem cells. Apoptosis is a part of the normal life of cells. By exploring the apoptosis mechanism of tumor cells and inducing the apoptosis, new treatment strategies can be developed.^41^

As a member of the PDCD10 family, PDCD10 plays an important role in many diseases.^14^, ^16^ It can either inhibit apoptosis or proliferation under different conditions and affect angiogenesis,^12^ but the definite regulatory mechanism in osteosarcoma is still unclear.

In this study, it was found that the expression of PDCD10 was increased in the tumor tissue of patients with osteosarcoma through bioinformatics analysis. The clinical immunohistochemical results indicated that the positive rate was high in patients with osteosarcoma, and it had a great correlation with prognosis. Therefore, we constructed PDCD10 knockdown cell lines with U2OS and MG63 cells and conducted a series of tumor cell function tests. The results illustrated that PDCD10 knockdown could significantly inhibit cell proliferation, migration, and invasion. In order to further verify its accuracy, we constructed PDCD10 overexpressing cell lines and repeated the relevant cell function tests. The results proved that PDCD10 overexpression can significantly promote cell proliferation, migration, and invasion. Afterward, we performed subcutaneous tumorigenesis assays in animals, and the results were consistent with the previous cellular assays. Tumors injected with PDCD10 overexpressing cells had significantly increased tumor size and weight. Through bioinformatics analysis, we predicted that PDCD10 may affect related signaling pathways in tumor progression. Based on WB results, PDCD10 could inhibit apoptosis and activate the EMT pathway.

At present, relative researches on PDCD10 mainly focus on tumors, and the findings have confirmed that PDCD10 can promote the process of tumor.^42^, ^43^ But in osteosarcoma, there are no related reports. Compared with previous research findings, in this study, we found that the positive rate of PDCD10 was high in patients with osteosarcoma. Among them, the five‐year mortality rate of PDCD10‐positive patients was also much higher than that of negative patients., which could be used as an important indicator to predict the treatment efficacy and five‐year survival rate in future clinical work. We also found that, in osteosarcoma, PDCD10 could inhibit tumor apoptosis and activate the EMT pathway to promote tumor progress.

The biggest flaw of this study is that the downstream genes and more detailed molecular mechanisms affected by PDCD10 have not been explored. Although an animal model of subcutaneous tumorigenesis has been constructed to prove its function, no animal assays of metastasis of osteosarcoma have been carried out, and the demonstration of the ability of PDCD10 to metastasize in osteosarcoma is insufficient.

## 5 CONCLUSION

In this study, it was found that PDCD10 was highly expressed in patients with osteosarcoma, and it was closely related to patient prognosis. The results indicated that the positive rate of PDCD10 was high in patients with osteosarcoma. Among them, the five‐year mortality rate of PDCD10 positive patients was also much higher than that of negative patients. These findings could be used to predict the treatment efficacy and five‐year survival rate of patients with osteosarcoma during clinical treatment and prognosis. PDCD10 inhibited apoptosis and activated the EMT pathway in promoting proliferation, migration, and invasion of osteosarcoma, which may provide a potential target for future bone tumor therapy.

## AUTHOR CONTRIBUTIONS

Ke Xu and Wenchao Fei: Conception, design, and operation for the work. Ziqi Huo and Shuoer Wang: Contributions to the conception and operation of the work. Yinghua Li: Data analysis and graphing. Gong Yang and Yang Hong: Funding acquisition and supervision of work. Paper writing, review, and editing for the work.

## FUNDING INFORMATION

This study was supported by Shanghai Science and Technology Commission Outstanding Academic Leader project for Yang Hong (20XD1402770); Minhang District Natural Science Foundation Project for Yang Hong (2019MHZ071); Special Project of Clinical Research in Health Industry of Shanghai Municipal Health Commission for Yang Hong (201940338); Research topics in the hospital for Yang Hong (2019WYZD01).

## CONFLICT OF INTEREST

The authors declared no competing interests.

## ETHICS APPROVAL

The approval of this study was authorized by the Fifth People's Hospital of Shanghai, Fudan University, China. Its registration number was AR2020141 and the examination number was m20200903.

## Data Availability

The data that support the findings of this study are available from the corresponding author upon reasonable request.
